# Artificial intelligence for detecting acute heart failure on chest CT: prospective clinical proof-of-concept validation

**DOI:** 10.1186/s41747-026-00718-x

**Published:** 2026-04-27

**Authors:** Kristina Cecilia Miger, Silas Nyboe Ørting, Anne Sophie Overgaard Olesen, Johannes Grand, Mikael Ploug Boesen, Michael Brun Andersen, Jens Petersen, Jens Jakob Thune, Marleen de Bruijne, Olav W. Nielsen

**Affiliations:** 1 Department of Cardiology, Copenhagen University Hospital—Bispebjerg and Frederiksberg, Copenhagen, Denmark; 2 Department of Clinical Medicine, University of Copenhagen, Copenhagen, Denmark; 3 Department of Computer Science, University of Copenhagen, Copenhagen, Denmark; 4 Department of Cardiology, Copenhagen University Hospital—Amager and Hvidovre, Copenhagen, Denmark; 5 Department of Radiology, Copenhagen University Hospital—Bispebjerg and Frederiksberg, Copenhagen, Denmark; 6 Department of Radiology, Copenhagen University Hospital—Herlev Gentofte, Copenhagen, Denmark; 7 Department of Radiology and Nuclear Medicine, Erasmus MC—University Medical Center Rotterdam, Rotterdam, The Netherlands

**Keywords:** Artificial intelligence, Dyspnea, Heart failure, Machine-learning, Tomography (x-ray computed)

## Abstract

**Objective:**

Acute heart failure (AHF) is a common but underrecognized cause of dyspnea. Chest computed tomography (CT) can accurately assess pulmonary congestion, but radiologist reporting capacity may limit clinical utility. We hypothesized that an artificial intelligence (AI) model could automatically detect imaging signs of AHF and aimed to prospectively validate an AI model in an independent emergency department cohort, benchmarking its performance against radiologists and cardiologists.

**Materials and methods:**

We prospectively validated a supervised machine-learning model in a single-center study of dyspneic patients undergoing low-dose, non-contrast chest CT and echocardiography. The primary analysis assessed diagnostic performance for CT-detected pulmonary congestion compatible with AHF, using radiologist-reported AHF as the reference and the area under the curve at receiver operating characteristic analysis (AUROC). Secondary analyses compared the AI model with blinded research radiologists and expert cardiologists.

**Results:**

Of 234 patients (56% males), aged 74 ± 10 years (mean ± standard deviation), 61 (26%) had radiologist-reported AHF. The AI model achieved high diagnostic performance (AUROC 0.95 [95% confidence interval 0.93–0.98]), with 89% sensitivity [78–95] and 89% specificity [83–93]. At prespecified thresholds, rule-out maximized sensitivity (97% [89–100]) at the expense of specificity (74% [67–81]), whereas rule-in yielded high specificity (96% [92–98]) but lower sensitivity (66% [52–77]). In secondary analyses, the AI model achieved a median AUROC of 0.94 (range 0.91–0.96).

**Conclusion:**

The AI model demonstrated high diagnostic performance for detecting AHF on chest CT in dyspneic patients. Integration into emergency workflows may support more consistent diagnosis, independent of clinician experience or time constraints.

**Relevance statement:**

AI-based analysis of chest CT may enable earlier and more consistent detection of AHF, supporting timely triage and management, especially when specialist radiological expertise is limited or delayed.

**Key Points:**

- An AI model prospectively detected AHF on chest CT in dyspneic emergency department patients.
- In a prospective single-center cohort, AI achieved high diagnostic performance (AUROC 0.91–0.96), comparable to that of radiologists and cardiologists.
- AI-based chest CT interpretation may improve diagnostic consistency in the absence of standardized CT criteria for AHF.

**Graphical Abstract:**

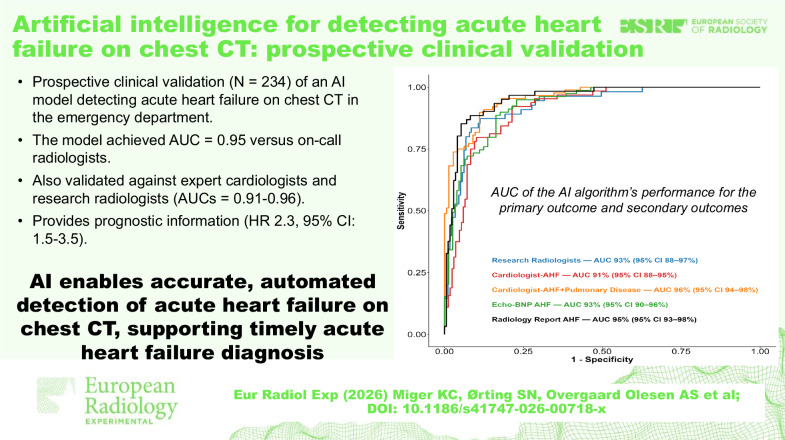

## Background

Acute heart failure (AHF) is a common cause of dyspnea in the emergency setting, posing a significant challenge in health care, with up to a third of hospitalized patients with AHF facing readmission or death within 3 months [1, 2]. Despite advancements in other cardiovascular fields, the outcomes for AHF remain disproportionately poor [3].

Dyspnea – a key symptom of AHF – stands as one of the most prevalent and distressing symptoms prompting patients to seek urgent medical attention [4]. During early triage, chest computed tomography (CT), the reference imaging modality for thoracic evaluation, is increasingly used in patients with undifferentiated dyspnea [5], as it provides comprehensive thoracic assessment with high spatial resolution, supports differential diagnosis [6], and is superior to chest radiography for detecting AHF [7] with acceptable radiation exposure [8]. Nevertheless, the wider use of CT in this setting is restricted by high radiology reporting workload and time-critical, high-pressure emergency conditions, which may delay integration into routine pathways.

Artificial intelligence (AI) has emerged as a potential adjunct for identifying critical diagnostic findings, with the aim of supporting, rather than replacing, radiologists. While we previously developed and retrospectively internally validated an interpretable AI model for detecting radiological signs of AHF on chest CT [9], the model has not been prospectively evaluated in consecutive acute patients presenting with undifferentiated dyspnea using prespecified thresholds and independent validation data.

We hypothesized that an AI-enabled CT interpretation can detect and triage for radiologic imaging signs consistent with AHF on chest CT in emergency patients presenting with dyspnea, with diagnostic accuracy comparable to radiologists and cardiologists with various clinical expertise. Our aim was to validate an in-house developed AI model for the detection of radiologic signs indicative of AHF on chest CT in consecutive adult patients admitted to the emergency department with dyspnea. Additionally, in exploratory analyses, we aimed to assess the prognostic value of the AI model in this population.

## Methods

The study was approved by the Danish National Committee on Health Research Ethics (H-17000869). All patients provided written informed consent.

### Study design and cohorts

**Fig. 1:**
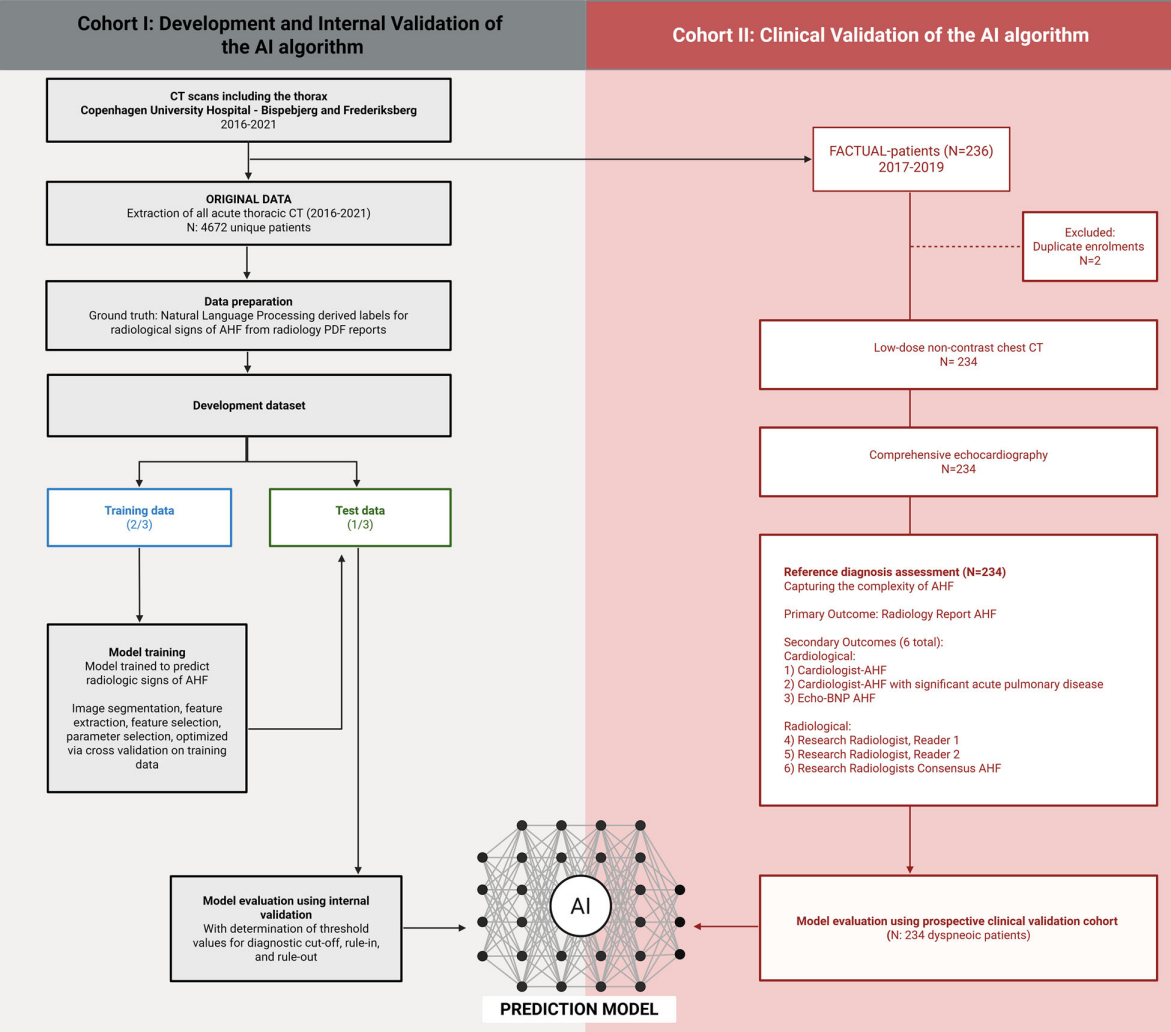
Flowchart illustrating the development and internal validation (gray background), and prospective clinical validation (red background) of the AI algorithm, including dataset inclusion, model training, and validation procedures. The number of patients included at each stage is indicated

This study comprised two sequential phases: (1) retrospective model development with internal validation using acute chest CT examinations acquired for any clinical indication, as previously described [9], and (2) independent prospective clinical validation in consecutive emergency department patients with acute dyspnea (Fig. 1). The present manuscript reports the independent prospective clinical validation of a previously developed AI model.

During model development [9], the retrospective cohort was split at the subject-level into training and internal validation datasets, with one-third reserved for internal validation [9]. The prospective cohort (Feasibility of CT in Patients with Acute Decompensated Heart Failure [FACTUAL]) was fully independent and used exclusively for clinical validation of the model.

### The primary outcome was the diagnostic performance of radiologic signs of AHF on chest CT

Secondary outcomes included alternative radiological and cardiological reference definitions and an exploratory longitudinal mortality analysis.

### Development and testing of the AI model

The methodology for the development and testing of the AI model has previously been described in detail [9]. In brief, the AI model was developed in patients who had a chest CT scan performed for any acute indication, encompassing a range of CT scanners using both high-dose, and low-dose protocols, as well as contrast-enhanced and non-contrast examinations. Ground truth for model development comprised radiological findings of AHF and pulmonary congestion extracted via text-mining of radiology reports [9].

The AI model was developed through parameter tuning and feature selection done on the training cohort only, using cross-validation at the subject level. TotalSegmentator [10] and XGBoost [11] were employed for anatomical segmentation and AHF prediction. Parameter tuning was done on the retrospective training data using grid search and cross-validation to compute metrics. Prevalence in the training data was around 7.4%. This imbalance was handled by including a scaling parameter in the tuning, which was set to either no scaling or scaling based on group size. The parameter tuning resulted in no scaling in the final model. Features that could not be computed were set to NaN and handled in the model by allowing it to make decisions, *i.e.*, branch in a tree, based on missing values.

The final AI model included 12 discriminating features [9]

1. mean density (HU) of lung boundary, defined as the 10-mm band at the edge of the lung (HU);
2. pleural ratio (pleural effusion volume/total lung volume);
3. left atrial volume;
4. pleural effusion volume;
5. median lung density (HU);
6. mean density of vena cava inferior (HU);
7. right atrial volume;
8. right ventricle ratio (right ventricle/total heart volume);
9. absolute heart *Z* score;
10. age;
11. right atrial density (HU); and
12. diameter of the inferior vena cava.

The absolute heart *Z* score was calculated as:

$$0.6745^{*} ({{\rm{V}}}{\_}{{\rm{patient}}}\; {{\rm{minus}}}\; {{\rm{V}}}{\_}{{\rm{train}}})/{{\rm{MAD}}}{\_}{{\rm{train}}}$$

where V_patient was the patient’s heart volume, V_train was the median heart volume of patients in training data, and MAD_train was the median absolute deviation from the median heart volume in training data.

The model included age as a clinical prior. Sex was evaluated but not retained, and additional demographic variables could not be assessed due to the use of pseudonymized data during model development [9]. The retrospective training data included contrast-enhanced and non-contrast scans; contrast-derived density features were zeroed in non-contrast scans to prevent contribution to the non-contrast validation cohort.

During development, the AI model was internally validated using a held-out test set comprising 1/3 of the retrospective cohort that was not used for training, achieving an AUROC of 0.87 and an optimal diagnostic threshold (cutoff = 0.064) to detect radiologic signs of AHF with the highest combination of both sensitivity and specificity. Similarly, thresholds for rule-in (cutoff = 0.026) and rule-out (cutoff = 0.150) were defined at a true positive rate = 90%, and true negative rate = 90%, respectively [9]. These internal validation results are reported for methodological completeness only and were published previously [9].

### Clinical validation cohort

We conducted a clinical validation of the AI model in a separate prospective observational cohort (FACTUAL) of consecutive patients presenting with dyspnea to the emergency department in the same hospital while ensuring that none of those patients or CT scans contributed to the development of the AI model (Fig. 1) [7, 12]. The study protocol has been previously published [12]. To ensure independence between cohorts, FACTUAL participants used for clinical validation had been removed a priori at the subject-level, together with every associated examination, before development of the AI model [9]. The AI model was applied without retraining or recalibration in the validation cohort, using the prespecified thresholds defined during model development [9].

The main inclusion criteria for the FACTUAL participants were admission to the emergency department at Copenhagen University Hospital—Bispebjerg and Frederiksberg, for dyspnea supported by at least one abnormal respiratory parameter (Supplemental Table S1), and age = 50 years. Patients were screened during 216 random weekdays from November 2017 to August 2019. The main exclusion criterion was if the CT or echocardiography could not be performed within 12 h [7, 13].

### Low-dose non-contrast chest CT

Non-contrast chest CT was performed using a multislice scanner (Somatom Definitions Flash, Siemens Medical Solutions) with a low-dose protocol (1.3 mSv [min-max range 1.2–1.4 mSv]) [13].

Images were analyzed by one of 20 different on-call clinical radiologists as part of the clinical routine. The on-call radiologists had access to the medical record and previous radiology images, but did not have access to echocardiography results. All radiology reports were confirmed by at least one senior experienced radiologist, and the final adjudicated radiology report was used as a reference in the present analysis.

### Comprehensive echocardiogram

The echocardiography was performed by experienced cardiologists within a few hours of admission according to the European Society of Cardiology guidelines. The examination included a systematic evaluation of left ventricular filling pressure and diastolic dysfunction [14, 15]. All echocardiographic examinations were reviewed by two cardiologists. For full details, see Miger et al [7].

### Primary outcome

The primary outcome, denoted as “radiology report AHF”, was the approved final clinical radiology report from the on-call radiologists, confirmed by a senior radiologist, as it aligned best with routine clinical practice. No predefined imaging criteria for pulmonary congestion compatible with AHF were imposed, as the intent was to reflect real-world clinical interpretation, which may vary between radiologists and incorporates available clinical context (*e.g.*, age, sex, presenting symptoms, and laboratory data).

### Secondary outcomes

While recognizing that the final radiology report may incorporate clinical context and comprehensively address the complexity of AHF diagnosis, we defined six secondary outcomes spanning both radiological and cardiological domains indicative of AHF to evaluate robustness beyond routine reporting (Supplemental Material: Appendix 1). These outcomes were intentionally included as complementary reference definitions to evaluate the robustness of the AI model across alternative definitions of AHF, reflecting the heterogeneous and multifactorial nature of the syndrome and enhancing its clinical relevance. In this manuscript, CT findings reflect radiologic signs of pulmonary congestion compatible with AHF, “radiology report AHF” denotes the routine clinical radiology report impression of AHF (primary outcome), whereas “cardiologist-AHF” denotes clinically adjudicated AHF (secondary outcomes).

The secondary outcomes, defined in detail in Appendix 1, were as follows:

Secondary outcomes—cardiology assessment of AHF: adjudicated by two cardiologists (third in case of disagreement) using echocardiography and medical records, without direct evaluation of CT images. Full operational details are provided in Appendix 1 [16].

1. Cardiologist-AHF: clinical diagnosis based on integrated assessment of symptoms, echocardiographic findings, biomarkers, and treatment response, without acute pulmonary disease.
2. Cardiologist-AHF with significant acute pulmonary disease: defined as cardiologist-AHF with concurrent clinically significant acute pulmonary disease (*e.g.*, pneumonia or acute exacerbation of chronic obstructive pulmonary disease) contributing to dyspnea, and cardiogenic pulmonary edema was considered part of the AHF spectrum.
3. Echo-BNP AHF: This objective, operator-independent diagnosis was designed to mitigate potential bias from medical record review and was based solely on echocardiographic, biomarker (NT-proBNP), hemodynamic, and treatment-related criteria.

Secondary outcomes – Radiology assessment of AHF:

4) Research Radiologist, Reader 1
5) Research Radiologist, Reader 2
6) Research Radiologists Consensus AHF.

These secondary radiologic outcomes were included as methodological sensitivity analyses to evaluate AI performance against blinded, expert interpretations independent of clinical context. Four secondary outcomes are presented in the main text, with individual results for the two research radiologists detailed in the Supplemental Material. In contrast to the primary outcome, the two research radiologists were specialized in thoracic imaging and provided an independent review using predefined imaging features [13], blinded to all clinical information, including previous radiology, radiology reports, and echocardiographic findings. Their task was to assess specific radiological findings to ensure high-quality labeling, identifying, and categorizing radiological features. The presence of radiological signs of AHF by the research radiologists was determined by unanimous consensus on a high probability of pulmonary congestion compatible with heart failure [13].

### Statistical analysis

R version 4.2.2 [17] was used for all statistical analyses. Patient characteristics are reported as continuous variables mean (± one standard deviation) when normally distributed and as median [interquartile range] when distributions were skewed, and compared variables with Student *t*-test or Wilcoxon, as appropriate. Categorical variables are reported as absolute numbers (percentages), and we used the? 2-test or Fisher's exact test for comparisons. All tests were two-sided.

Performance measurement was evaluated using the area under the curve at receiver operating characteristic analysis (AUROC). We also demonstrated sensitivity, specificity, negative predictive value, positive predictive value, positive likelihood ratio, and negative likelihood ratio. This validation study used an existing prospective cohort with fixed enrollment, and precision is therefore communicated via 95% confidence intervals [CIs] for all performance estimates.

To characterize the subset of patients with inaccurate AI predictions and to categorize the features associated with AI errors, we identified both false positive and false negative cases. False positive cases were defined as AI-predicted radiological signs of AHF not confirmed by the final radiology report, whereas false negative cases were defined as AI-negative predictions despite radiological signs of AHF reported by the radiologists.

Follow-up time was measured from study enrollment until death or the end of follow-up. Mortality data were obtained from electronic medical records. Survival was analyzed using the Kaplan–Meier method and Cox proportional hazards models.

## Results

### Patient characteristics

All results presented below refer to the prospective clinical validation cohort. Model development and internal validation were performed in a separate retrospective cohort and are reported elsewhere [9]. In the prospective clinical validation cohort, 236 patients admitted with acute dyspnea were initially enrolled in the study. However, two patients had duplicate enrollments, resulting in a final sample size of 234 patients. Among these, 61 patients (26%) met the primary outcome, radiology report AHF, as reported by the on-call radiologists. Patients with radiology report AHF, compared to patients without, were significantly older, 44% had a history of heart failure, they exhibited less chronic obstructive pulmonary disease, higher systolic blood pressure at admission, and more symptoms of heart failure such as orthopnea, bilateral rales on auscultation, and bilateral pedal edema (Table 1). Furthermore, patients with the primary outcome had higher NT-proBNP levels and more abnormal echocardiographic parameters (Table 1).

**Table 1:** Characteristics of the patient population according to the primary outcome, radiologic AHF, identified by the on-call clinical radiologists

	All	No AHF on the radiology report	AHF on radiology report	*p*-value
	*n* = 234	*n* = 173	*n* = 61	
Medical history				
Age, mean (SD)	74.2 (10.1)	72.7 (9.9)	78.4 (9.7)	< 0.001
Male, *n* (%)	131 (56.0)	93 (53.8)	38 (62.3)	0.315
Chronic heart failure, *n* (%)	58 (24.8)	31 (17.9)	27 (44.3)	< 0.001
Chronic obstructive pulmonary disease, *n* (%)	122 (52.1)	108 (62.4)	14 (23.0)	< 0.001
Diabetes mellitus (type I and II), *n* (%)	58 (24.8)	37 (21.4)	21 (34.4)	0.064
Hypercholesterolemia, *n* (%)	70 (29.9)	47 (27.2)	23 (37.7)	0.167
Known kidney disease, *n* (%)	22 (9.40)	14 (8.09)	8 (13.1)	0.368
Hypertension, *n* (%)	144 (61.5)	100 (57.8)	44 (72.1)	0.068
Signs and symptoms at admission				
Body mass index (kg/m^2^), median [IQR]	25.8 [22.7, 20.0]	26.0 [22.7, 30.1]	25.5 [23.4, 28.8]	0.947
Systolic blood pressure (mmHg), mean (SD)	143 (27.8)	140 (25.6)	154 (30.9)	0.002
Orthopnea, *n* (%)	113 (48.3)	69 (39.9)	44 (72.1)	< 0.001
Bilateral pedal edemas, *n* (%)	71 (30.3)	40 (23.1)	31 (50.8)	< 0.001
Bilateral rales, *n* (%)	88 (37.6)	47 (27.2)	41 (67.2)	< 0.001
Laboratory data				
Abnormal ECG, *N* (%)	107 (45.7)	59 (34.1)	48 (78.7)	< 0.001
eGFR, median [IQR]	69.0 [49.0; 84.8]	75.0 [52.0; 88.0]	57.0 [46.0; 78.0]	0.006
NT-proBNP (pg/mL), median [IQR]	867 [231; 3,273]	496 [141; 1,911]	3,645 [2,055; 6,698]	< 0.001
Echocardiographic data				
LVEF, median [IQR]	55.0 [45.0; 60.0]	60.0 [51.0; 60.0]	40.0 [25.0; 50.0]	< 0.001
Echocardiographic phenotype, *n* (%)				
Reduced LVEF = 40%	52 (22.2)	21 (12.1)	31 (50.8)	< 0.001
Mildly reduced LVEF (41–49%)	24 (10.3)	14 (8.1)	10 (16.4)	0.111
LVEF = 50% with diastolic dysfunction	47 (20.1)	28 (16.2)	19 (31.1)	0.020
Severe valve disease	9 (3.85)	4 (2.31)	5 (8.2)	0.054
E/e’, median [IQR]	10.5 [7.90; 14.6]	8.95 [7.27; 11.7]	17.2 [13.1; 22.8]	< 0.001
Indexed left atrial volume (mL/m^2^), mean (SD)	34.4 (15.7)	30.0 (14.5)	46.7 (12.0)	< 0.001
Elevated filling pressure (grade II + III), *n* (%)	92 (39.3)	33 (19.1)	59 (96.7)	< 0.001

*ECG* Electrocardiography, *eGFR* Estimated glomerular filtration rate, *LVEF* Left ventricular ejection fraction, *NT-proBNP* N-terminal pro-B-type natriuretic peptide, *SD* Standard deviation

Regarding the secondary outcomes, cardiologist-AHF was adjudicated in 64 patients (27%), with an additional 24 patients (10%) having AHF combined with significant acute pulmonary disease, yielding a total of 88 patients (37%) with any cardiology-adjudicated AHF. Echo-BNP AHF was identified in 80 patients (34%). For the secondary radiological outcome, Research Radiologists Consensus AHF, was observed in 55 (24%) patients. Results for research radiologists 1 and 2 are presented individually in the Supplemental Fig. S1.

### AI in action: detecting AHF

**Fig. 2:**
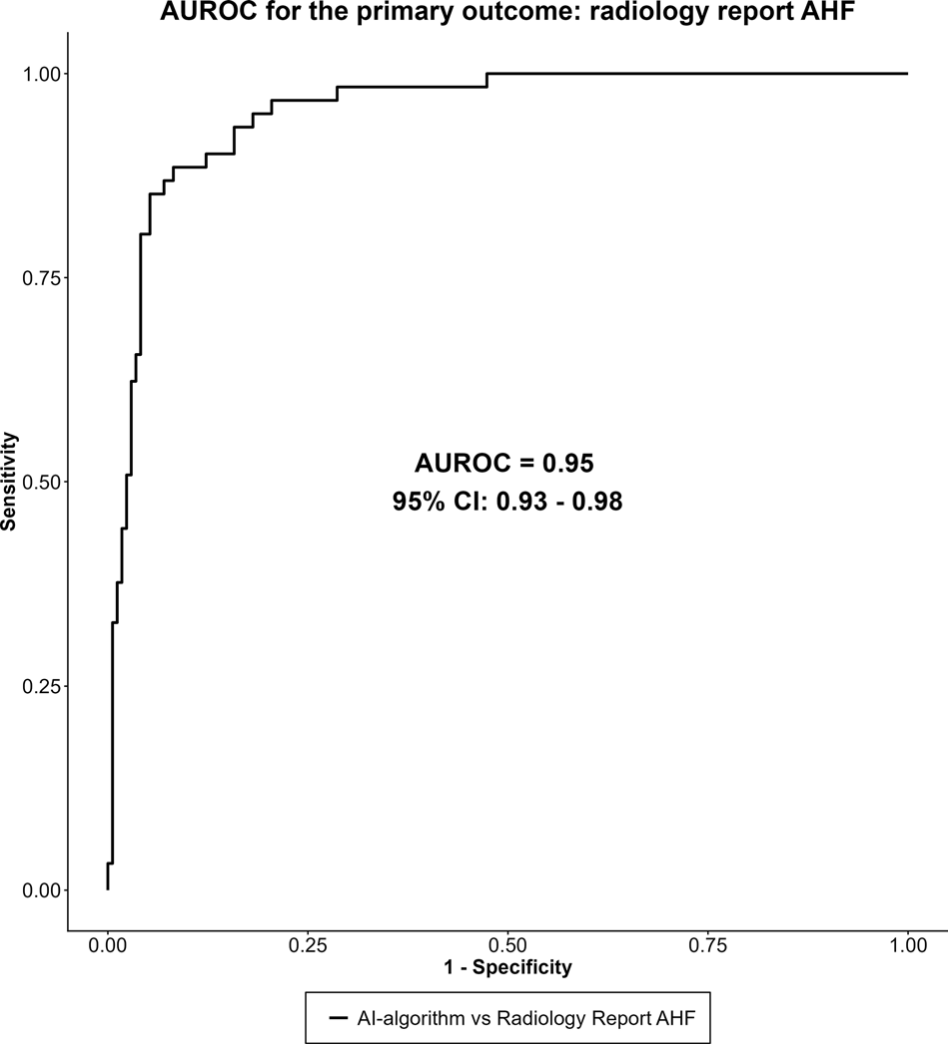
Area under the receiver operating characteristic curve (AUROC) of the AI algorithm for the primary outcome, *i.e.*, the report of AHF from the on-call radiologists, confirmed by the senior radiologist, and served as the reference standard. AUROC values are reported with 95% CIs

**Fig. 3:**
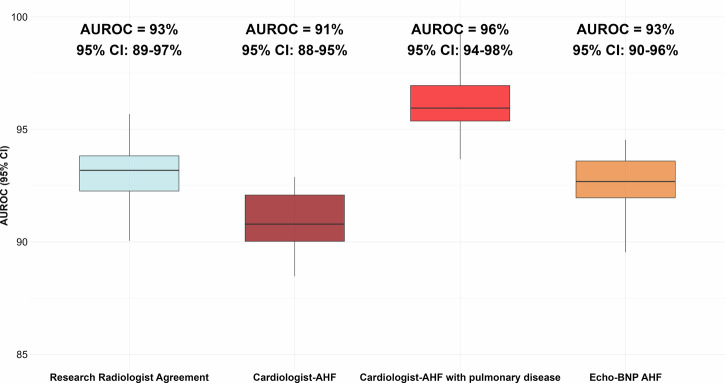
Boxplots illustrating the AUROCs for the AI across secondary outcomes. The reference standards for the secondary outcomes were as follows: (1) agreement between two independent, blinded thoracic research radiologists for AHF (light blue); (2) cardiologist-adjudicated AHF without concomitant pulmonary disease (dark red); (3) cardiologist-adjudicated AHF with concomitant significant acute pulmonary disease (light red); and (4) observer-independent AHF diagnosis based on echocardiography, B-type natriuretic peptide levels, and relevant treatment (Echo-BNP) (orange). Whiskers indicate the 95% CI of the AUROC estimates

The AI model successfully generated AHF predictions for 232 of 234 patients (99%), where the AI model detected the primary outcome, radiology report AHF, with an AUROC of 0.95 (95% CI: 0.93–0.98) (Fig. 2). Two predictions could not be generated due to an error in data extraction from the Picture Archiving and Communication System. Consistent results were illustrated for the secondary outcomes, with a median AUROC of 0.94 and a range between 91% and 96% (95% CIs: 88–98%, Fig. 3 and Supplemental Fig. S1).

Using the predefined diagnostic threshold with the highest combination of both sensitivity and specificity, the AI model demonstrated robust performance in detecting the primary outcome with a sensitivity of 89% (95% CI: 78%–95%) and a specificity of 89% (95% CI: 83%–93%) (Table 2). Employing the prespecified rule-out threshold from the model derivation, the AI model yielded a sensitivity of 97% (95% CI: 89%–100%), and a specificity of 74% (95% CI: 67%-81%) (Table 2). Similarly, for the prespecified rule-in threshold, the AI model yielded a specificity of 96% (95% CI: 92%–98%), and a sensitivity of 66% (95% CI: 52%–77%) (Table 2). The calibration characteristics are shown in Supplemental Fig. S2.

**Table 2:** Diagnostic accuracy table for the performance of our AI model to diagnose AHF using the primary outcome diagnosis

	Diagnostic cutoff	Rule-out	Rule-in
Sensitivity (%)	89	97	66
(95% CI)	(78–95)	(89–100)	(52–77)
Specificity (%)	89	74	96
(95% CI)	(83–93)	(67–81)	(92–98)
PPV (%)	74	57	85
(95% CI)	(62–84)	(47–67)	(72–94)
NPV (%)	96	98	89
(95% CI)	(91–98)	(95–100)	(83–93)
PLR	7.97	3.76	16.02
(95% CI)	(5.16–12.29)	(2.90–4.87)	(7.58–33.84)
NLR	0.13	0.04	0.36
(95% CI)	(0.06–0.26)	(0.01–0.17)	(0.25–0.51)

Predefined rule-out, rule-in, and diagnostic cutoff values established from model evaluation during internal validation were used. Reference standard for the primary outcome: AHF radiology report

*CI* Confidence interval, *NLR* Negative likelihood ratio, *NPV* Negative predictive value, *PLR* Positive likelihood ratio, *PPV* Positive predictive value

### Characterizing misclassified cases: overlooked and overcalled AHF

At the predefined diagnostic threshold, the AI model classified 19 (8%) CT scans as false positives and 7 (3%) as false negatives; all seven were adjudicated as AHF by the cardiologists. The false positive predictions were primarily associated with acute pulmonary disease, whereas false negatives represented confirmed AHF with less pronounced radiologic signs of congestion (Supplemental Table S2 and Supplemental Fig. S3).

### Prognostic association of AI-defined AHF

**Fig. 4:**
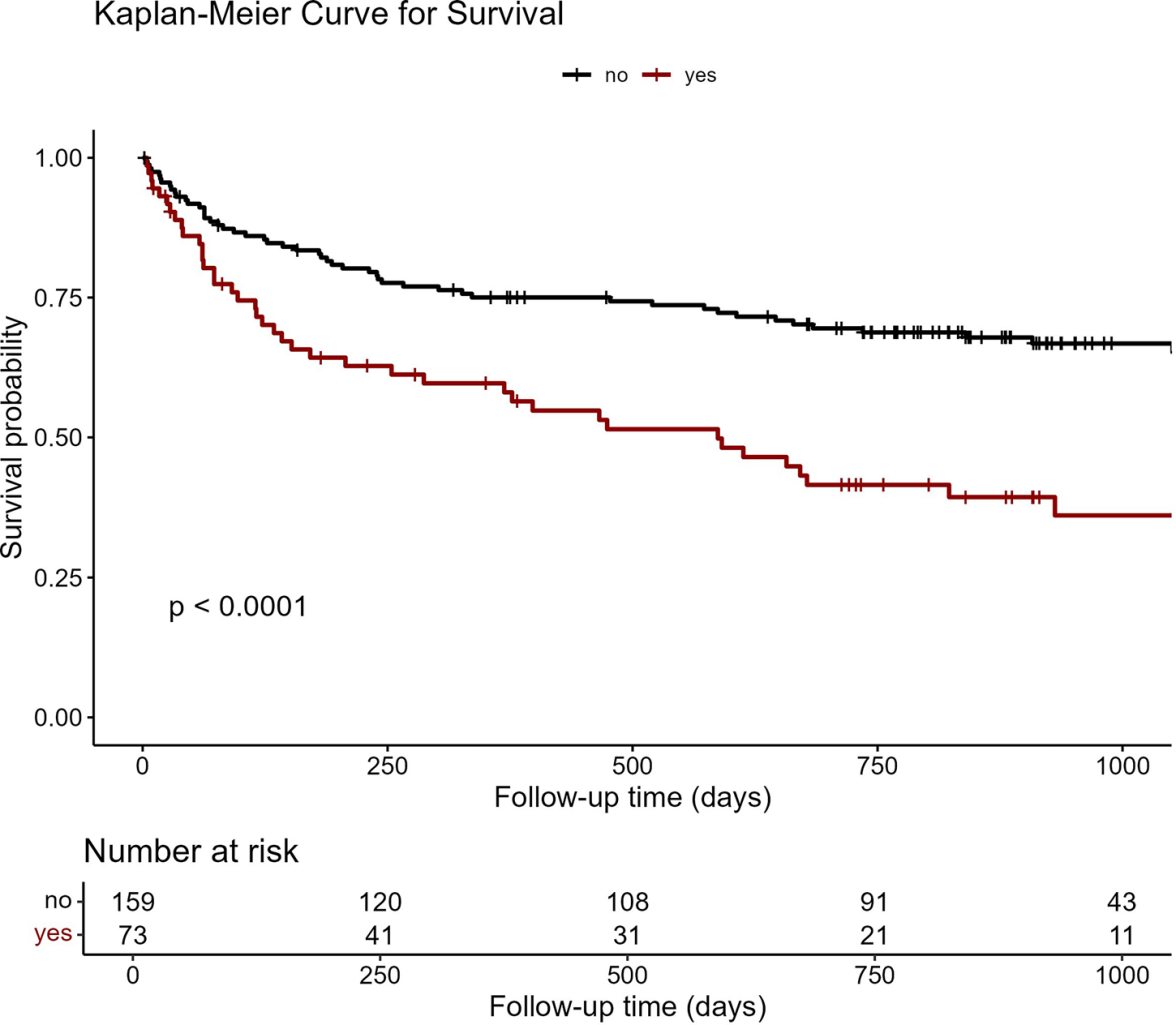
Kaplan–Meier survival curves stratified by AI classification of AHF. Patients classified as AHF-positive by the AI model (red) are compared with those classified as AHF-negative (black). AHF classification was based on the predefined diagnostic threshold defined in the Methods section. Survival was assessed over a follow-up period of 2.0 years. Group differences were evaluated using the log-rank test (*p* < 0.0001), with corresponding hazard ratios and 95% CIs reported in the “Results” section.

The median follow-up time was 2.0 years (95% CI: 1.8–2.2). In the exploratory survival analyses, the AI output was prognostically meaningful. Patients classified as having AHF by the prespecified diagnostic threshold showed worse unadjusted survival (hazard ratio 2.3, 95% CI: 1.5–3.5; *p* < 0.001; Fig. 4). However, after adjusting for age, sex, and NT-proBNP, the association with mortality was no longer statistically significant (hazard ratio 1.10, 95% CI: 0.68–1.80; *p* = 0.686).

## Discussion

**Fig. 5:**
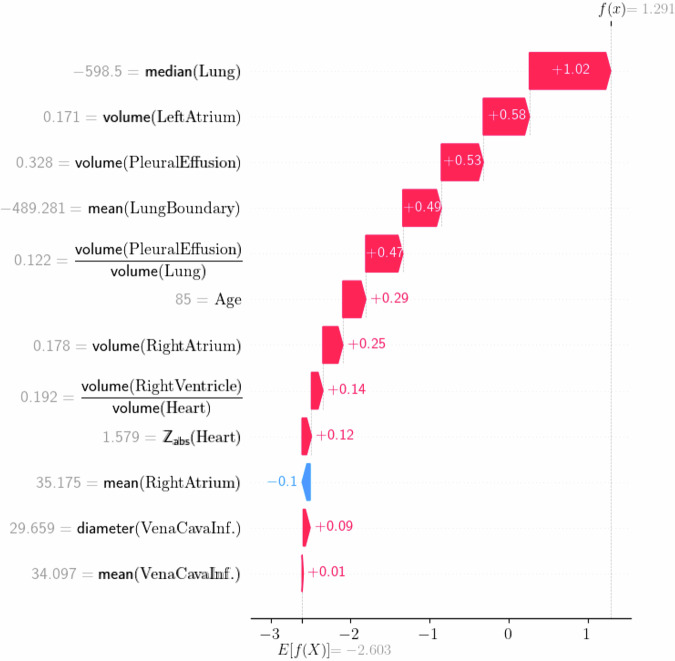
Illustrative example of Shapley Additive exPlanations (SHAP) values for a randomly selected patient with AHF as reported in the approved final clinical report from the on-call radiologists, confirmed by a senior radiologist) and a corresponding AI model prediction. Red arrows pointing to the right indicate features that increase the likelihood of AHF, whereas blue arrows pointing to the left indicate features that decrease it. The arrow length and SHAP values represent the contribution of each feature to the final prediction of the model output (log-odds) scale

This study highlights the potential of AI to support the identification of radiological signs of AHF on chest CT among those presenting with acute dyspnea. The AI model achieved excellent agreement with both radiologic and clinical reference diagnoses, with balanced sensitivity and specificity of 89% at the prespecified diagnostic threshold and expected trade-offs at rule-in and rule-out thresholds. Its performance was consistent across multiple reference standards for AHF (AUROCs from 0.91 to 0.96). Notably, for radiologists, AI may contribute to greater consistency in diagnosing pulmonary congestion, as there is currently no universally standardized approach for identifying CT findings indicative of AHF. Fig. 5.

### Existing evidence on the Use of AI for detecting AHF on chest CT

AI-based analysis of chest CT has been explored in multiple pulmonary and cardiovascular contexts [18–20]. However, to our knowledge, no prior studies other than our own have specifically reported prospective clinical validation of an AI model for the detection of radiological signs of AHF on chest CT. The present study is novel in that it provides prospective clinical validation of a previously developed AI model [9] in a consecutive emergency department cohort of patients with undifferentiated acute dyspnea, using an independent subject-level dataset with no overlap with model development and applying prespecified diagnostic, rule-in, and rule-out thresholds without retraining or recalibration. Beyond confirming discrimination performance, the study adds clinical relevance by benchmarking against routine on-call radiology reporting, as well as blinded expert radiologists and cardiology adjudication, systematically characterizing false-positive and false-negative cases in a real-world dyspnea population, thereby advancing the evidence from technical feasibility to workflow-relevant clinical validation, including longitudinal data.

While no prior studies have directly addressed AHF detection on chest CT, a limited number of related studies exist. Velichko et al reported that a machine learning algorithm could distinguish between COVID-19 pneumonia and pulmonary edema [21].

Jain et al demonstrated that machine learning-based lung water quantification can detect subclinical pulmonary congestion in patients with heart failure with a preserved ejection fraction [22], supporting the concept that AI-derived imaging features may capture early pathophysiological changes. Consistent with this, our algorithm achieved high sensitivity for detecting AHF, albeit with a concomitant decrease in specificity and an increase in false positives.

This study provides the first exploratory prognostic insights from an imaging-based AI model. Although the AI-defined AHF classification was associated with mortality in unadjusted analyses, this association was no longer significant after adjustment for age, sex, and NT-proBNP levels, indicating a substantial overlap with established clinical and biochemical risk markers. Consequently, these findings should be considered hypothesis-generating, requiring validation in larger cohorts. The potential value of such an imaging-based AI model may lie in complementing existing risk markers within a broader risk stratification framework, consistent with prior work demonstrating long-term cardiovascular risk prediction using automated CT-derived features [23].

### Challenges of the AI model

We took care to validate the algorithm on a separate patient cohort from that used for the development. The development cohort included all patients who had an acute CT scan independent of the presumed indication, while the clinical validation cohort in the present analysis consisted of patients with dyspnea included in the prospective FACTUAL study [7]. The development data included a broad mix of subjects from various internal medicine wards, encompassing diverse admission diagnoses, various CT scan models, protocols, reconstruction techniques, high- and low-dose protocols, and both contrast-enhanced and non-contrast scans. In contrast, the clinical validation cohort only included patients admitted to the emergency department with dyspnea as the sole diagnosis, all examined with a non-contrast, low-dose chest CT. Hence, although the population came from the same region with an overlapping temporal setting, the patient populations differed significantly, and stringency ensured there was no patient overlap.

We observed that the original development cohort for the AI model achieved an AUROC of 0.87 against the reference radiology AHF diagnosis [9]. However, the present prospective clinical validation cohort achieved significantly higher AUROCs across a range of reference diagnoses. We ascribe this higher performance to a greater homogeneity of the clinical cohort, as well as a higher quality of the radiologist-reported diagnoses, as they had a particular focus on describing heart failure.

### Rationale for multiple study outcomes

AHF is a complex clinical syndrome without a single universally accepted diagnostic gold standard. To reflect this complexity and to avoid over-reliance on a single reference definition, we intentionally evaluated AI performance across multiple radiological and cardiological outcome definitions. By doing so, the present study establishes a framework for future research to investigate whether imaging-based AI methods may also contribute to more objective or standardized definitions of heart failure, beyond conventional reporting paradigms.

The primary radiological endpoint was selected to reflect the AI model’s intended clinical role: providing automated decision support alongside routine radiologist interpretation. This could help support triage, prioritizing reading lists, and serving as a decision aid for non-radiologists before the final radiology report becomes available. However, routine clinical radiology reports may be influenced by clinical context, potentially affecting AI performance. To address this, we evaluated AI performance across multiple alternative reference standards as sensitivity analyses, including blinded research radiologist assessment, cardiology-adjudication independent of CT review, and an Echo-BNP–based definition. The consistently high performance across these increasingly objective reference standards (AUROCs from 0.91 to 0.96, with 95% CIs ranging from 88% to 98%) suggests that the diagnostic signal of the AI model is robust and not solely driven by alignment with routine clinical reporting.

### Practical considerations for clinical integration

In practice, the AI model could be integrated as an automated background analysis of chest CT examinations performed for acute dyspnea, without altering image acquisition or routine reporting. The model output could be displayed in the radiology worklist or report as a non-interruptive decision-support signal to support case prioritization, rather than as a standalone alert. SHapley Additive exPlanations-SHAP-based feature attribution may facilitate transparent case-level verification (Fig. 5). A potential benefit of this technology would be to assist younger and less experienced readers in responding more quickly to key findings, similar to prior emergency department decision-support applications [24, 25]. Prospective studies are required to assess the workflow integration, user acceptance, and clinical impact. Deployment would also require an appropriate regulatory pathway, either under the European Union Medical Device Regulation health-institution exemption for in-house use or, for broader implementation, Conformité Européenne-CE marking following multicenter clinical validation.

### Diagnostic errors: false positives and false negatives

Supplemental Table S2 summarizes the clinical characteristics of patients with false positive and false negative classifications. Using the applied diagnostic threshold may result in missed true heart failure cases and misclassification of patients with acute pulmonary disease as heart failure, reflecting the challenge of distinguishing cardiogenic from non-cardiogenic pulmonary abnormalities on CT. When clinical suspicion of AHF exists, echocardiography should not be delayed, even if radiological findings are negative, though access to acute echocardiography may vary across institutions. AI should be regarded solely as a supportive adjunct rather than a substitute for the comprehensive radiology report, which remains essential for assessing differential diagnoses.

### Study limitations

This study has several limitations. First, although validated in an independent cohort, it was conducted at a single center within a specific temporal and demographic setting, limiting generalizability. Second, the prospective validation cohort exclusively used low-dose, non-contrast chest CT and included patients aged =50 years or older, which may not reflect all emergency imaging pathways or younger populations. Despite differences in clinical indications, CT scanners, and acquisition protocols between cohorts, broader validation across diverse populations and health care systems is warranted. Differences in scanner type, acquisition parameters, and reconstruction parameters alter the image noise, noise texture, and attenuation within individual voxels, thereby affecting feature extraction. Variations in patient demographics (age and sex were available; other demographics, including ethnicity, could not be assessed due to pseudonymization during model development) and clinical workflows may influence anatomy, disease prevalence, and imaging phenotypes, thereby affecting the model’s generalizability and calibration. Third, the AI model was trained using routine emergency radiology reports, which may have introduced some degree of label variability related to the clinical context and acute workflow conditions. Fourth, our model depends on segmentations trained on human annotations without formal validation against expert references in our cohort. Segmentation inaccuracies may propagate to feature extraction, and systematic segmentation errors could affect performance; this was not explicitly quantified. Finally, diagnostic, rule-in, and rule-out thresholds were prespecified during model development and applied unchanged in the validation cohort as recalibration was intentionally avoided to prevent optimism bias. Threshold transportability requires further multicenter evaluation.

## Conclusions

We clinically validated an interpretable AI model that automatically identified radiologic signs of AHF on chest CT in patients presenting with dyspnea. The AI model demonstrated strong diagnostic performance for both radiology and cardiology definitions of AHF (AUROCs from 0.91 to 0.96) and provided mortality risk information complementary to NT-proBNP, offering timely risk information from routine CT without additional testing. Automated CT interpretation may serve as a decision-support tool to complement routine radiologist interpretation in patients presenting with dyspnea.

## Supplementary information

**Additional file 1**: **Table S1** Objective parameters supporting respiratory imbalance. **Fig. S1** The performance of the artificial intelligence algorithm in comparison to the secondary outcomes: research radiologist 1 (dark green) and research radiologist 2 (green). The two research radiologists independently identified radiologic signs of AHF in 60 patients (25%) and 64 patients (27%), respectively. **Fig. S2** Model calibration analysis. (**a**) Calibration plot in the independent prospective cohort showing suboptimal agreement of predicted probabilities and observed outcomes (Brier score 0.16). Vertical lines indicate the 95% binomial CIs for the observed event rates. (**b**) Calibration curves obtained by randomly splitting the FACTUAL data (*n* = 232) into two, calibrating the model on one part (*n* = 116) and predicting on the other part (*n* = 116). Each curve corresponds to one random split. The AUROC is provided in the legend. Recalibration improves agreement between predicted probabilities and observed outcomes, with substantial variability across splits reflecting the limited calibration sample size (*n* = 116). **Fig. S3**—Aggregated feature importance plots across the whole cohort (**a**), and stratified by true positives (**b**), false positives (**c**), and false negatives (**d**). **Table S2**—Patient characteristics for false positive cases and false negative cases. **Appendix 1**—Overview of the primary and secondary outcomes

## Data Availability

The data underlying this article cannot be shared publicly due to the privacy of the individuals who participated in the study. The source code for the AI model will be made publicly available at https://github.com/orting/breathct.

## Abbreviations

AHF: Acute heart failure
AI: Artificial intelligence
AUROC: Area under the curve at receiver operating characteristic analysis
BNP: B-type natriuretic peptide
CI: Confidence interval
CT: Computed tomography
FACTUAL: Feasibility of Computed Tomography in Patients with Acute Decompensated Heart Failure
NT-proBNP: N-terminal pro-B-type natriuretic peptide
