# Supplementary material for: Artificial intelligence for detecting acute heart failure on chest CT: prospective clinical proof-of-concept validation
Source: Eur Radiol Exp. 2026 Apr 27;10:55. doi: 10.1186/s41747-026-00718-x (PMC13121664; doi:10.1186/s41747-026-00718-x)
Supplement: Supplementary file 1 — Additional file 1: Table S1 Objective parameters supporting respiratory imbalance. Fig. S1 The performance of the artificial intelligence algorithm in comparison to the secondary outcomes: research radiologist 1 (dark green) and research radiologist 2 (green). The two research radiologists independently identified radiologic signs of AHF in 60 patients (25%) and 64 patients (27%), respectively. Fig. S2 Model calibration analysis. (a) Calibration plot in the independent prospective cohort showing suboptimal agreement of predicted probabilities and observed outcomes (Brier score 0.16). Vertical lines indicate the 95% binomial CIs for the observed event rates. (b) Calibration curves obtained by randomly splitting the FACTUAL data (n = 232) into two, calibrating the model on one part (n = 116) and predicting on the other part (n = 116). Each curve corresponds to one random split. The AUROC is provided in the legend. Recalibration improves agreement between predicted probabilities and observed outcomes, with substantial variability across splits reflecting the limited calibration sample size (n = 116). Fig. S3—Aggregated feature importance plots across the whole cohort (a), and stratified by true positives (b), false positives (c), and false negatives (d). Table S2—Patient characteristics for false positive cases and false negative cases. Appendix 1—Overview of the primary and secondary outcomes [file 41747_2026_718_MOESM1_ESM.pdf]

Artificial intelligence for detecting acute heart failure on chest CT: prospective clinical proof-of-concept validation

ELECTRONIC SUPPLEMENTARY MATERIAL

Table S1 Objective parameters supporting respiratory imbalance

Respiratory rate ≥2 0 breaths/minute
Saturation < 95% (if known chronic obstructive pulmonary disease, < 92%)
Abnormal pCO_2or pO_2 in arterial blood
Objective signs of heart failure (i.e., peripheral edema, jugular vein distention, orthopnea, bilateral rales on auscultation)
Prolonged breathing or rhonchi on auscultation

To support acute dyspnea at patient inclusion, at least one abnormal respiratory parameter was required

Fig. S1 The performance of the artificial intelligence algorithm in comparison to the secondary outcomes: research radiologist 1 (dark green) and research radiologist 2 (green). The two research radiologists independently identified radiologic signs of acute heart failure (AHF) in 60 patients (25%) and 64 patients (27%), respectively.

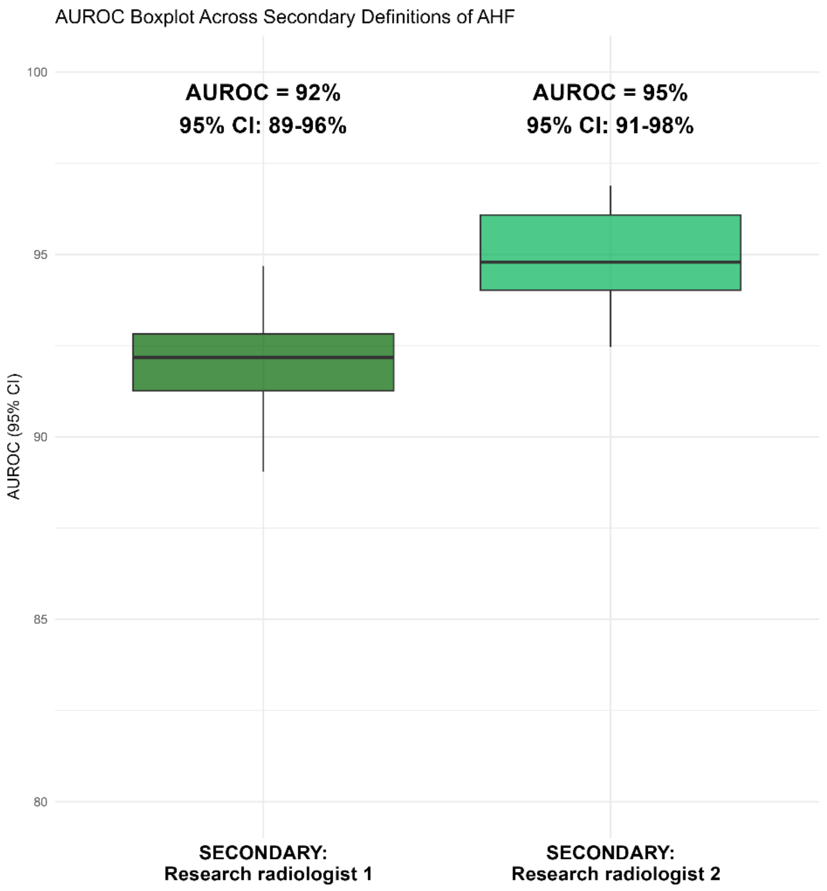

Fig. S2 Model calibration analysis. (**a**) Calibration plot in the independent prospective cohort showing suboptimal agreement of predicted probabilities and observed outcomes (Brier score 0.16). Vertical lines indicate the 95% binomial confidence intervals for the observed event rates.

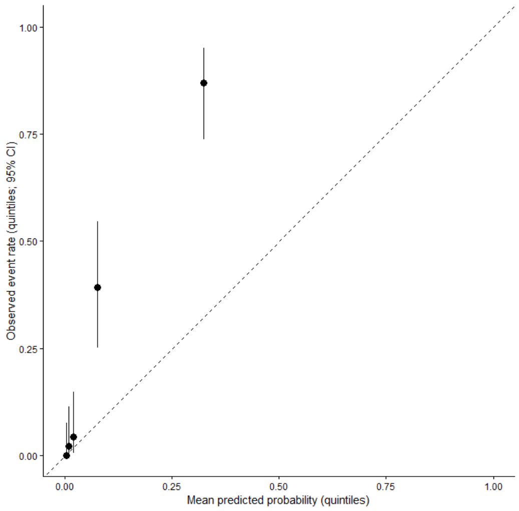

(**b**) Calibration curves obtained by randomly splitting the FACTUAL data (*n* = 232) in two, calibrating the model on one part (*n* = 116) and predicting on the other part (*n* = 116). Each curve corresponds to one random split. The AUROC is provided in the legend. Recalibration improves agreement between predicted probabilities and observed outcomes, with substantial variability across splits reflecting the limited calibration sample size (*n* = 116).

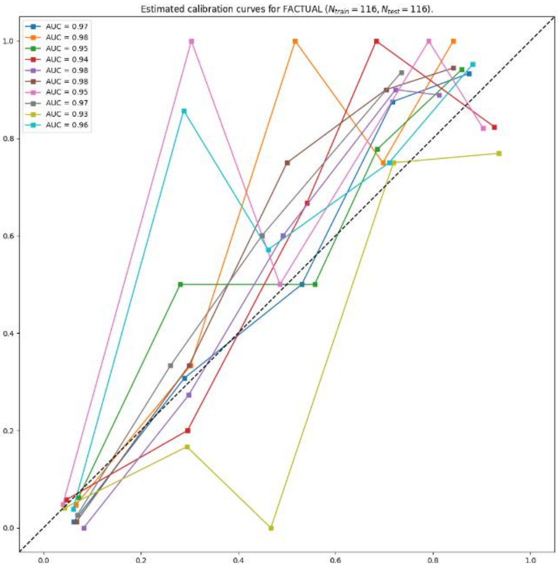

Fig. S3 - Aggregated feature importance plots across the whole cohort (**a**), and stratified by true positives (**b**), false positives (**c**), and false negatives (**d**).

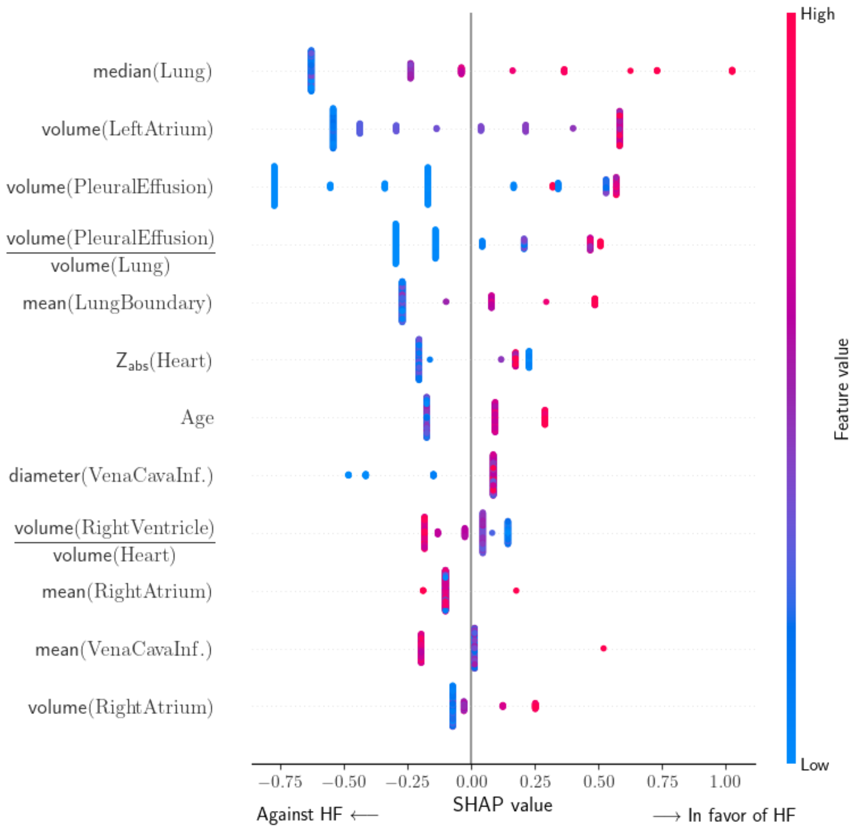

(a)

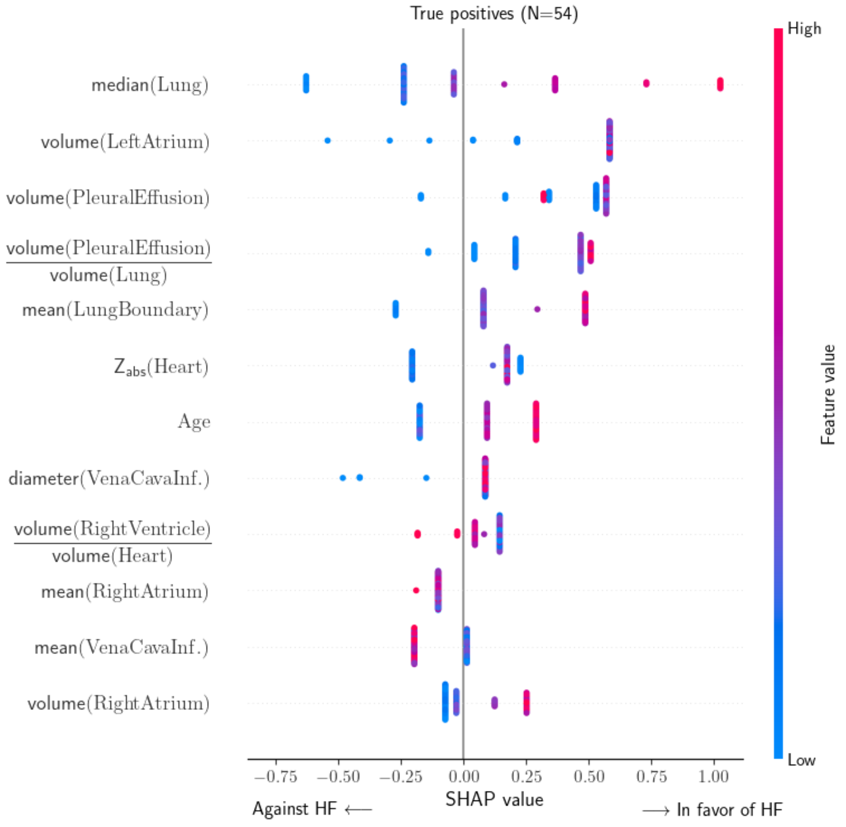

(b)

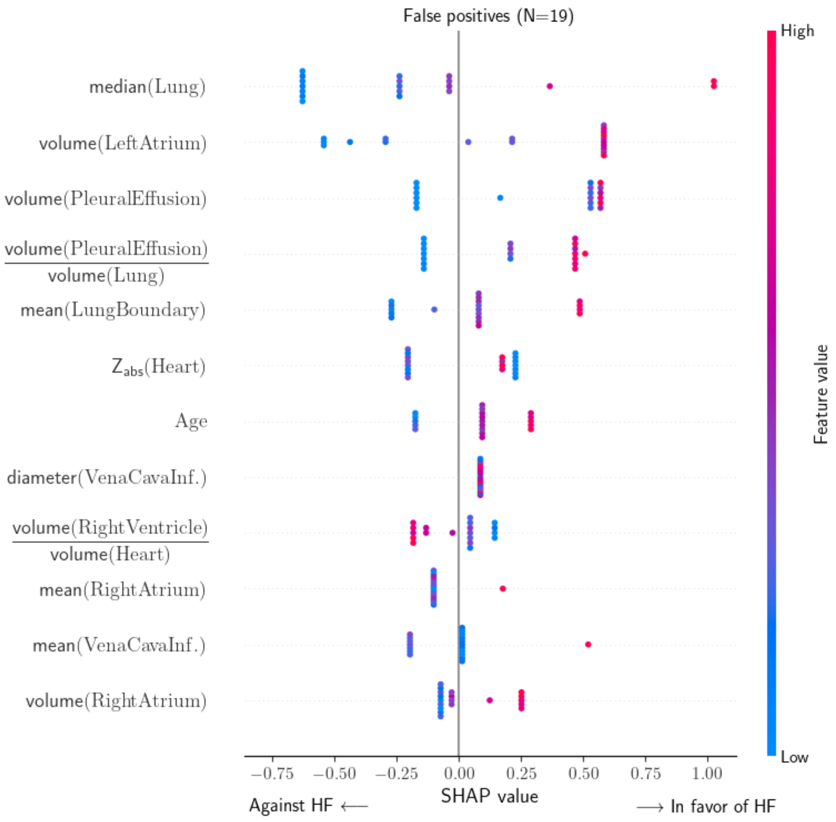
False positives (N=19)
median(Lung)
volume(LeftAtrium)
volume(PleuralEffusion)
volume(PleuralEffusion) / volume(Lung)
mean(LungBoundary)
Zabs(Heart)
Age
diameter(VenaCavaInf.)
volume(RightVentricle) / volume(Heart)
mean(RightAtrium)
mean(VenaCavaInf.)
volume(RightAtrium)
−0.75 −0.50 −0.25 0.00 0.25 0.50 0.75 1.00
SHAP value
Against HF ⟵
⟶ In favor of HF
High
Feature value
Low

(c)

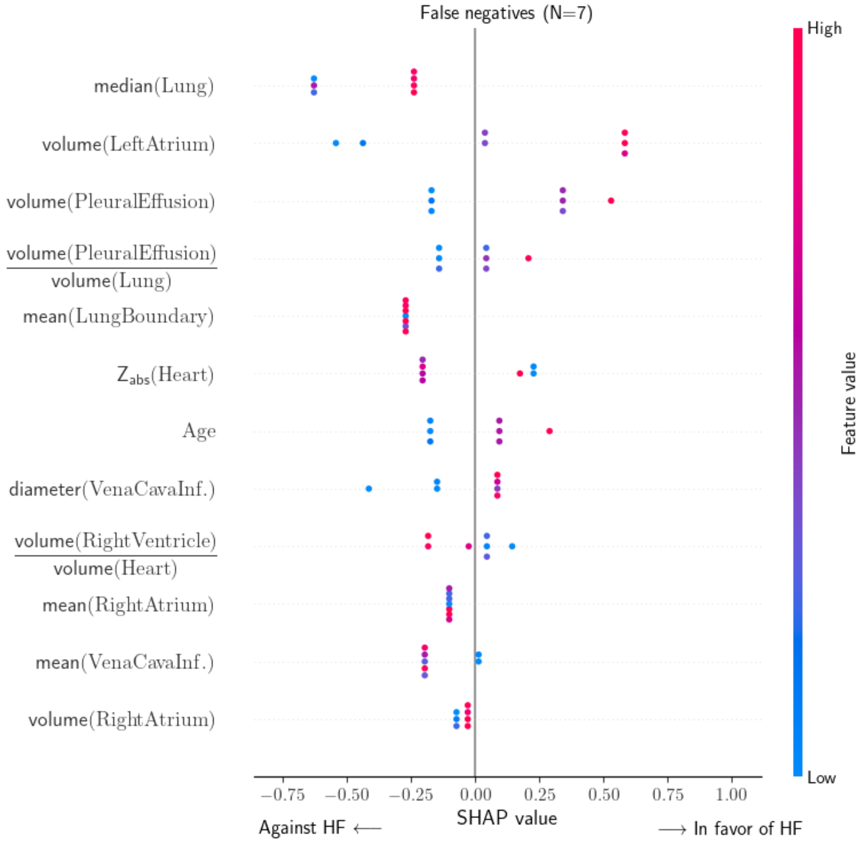
False negatives (N=7)
median(Lung)
volume(LeftAtrium)
volume(PleuralEffusion)
volume(PleuralEffusion) / volume(Lung)
mean(LungBoundary)
Zabs(Heart)
Age
diameter(VenaCavaInf.)
volume(RightVentricle) / volume(Heart)
mean(RightAtrium)
mean(VenaCavaInf.)
volume(RightAtrium)
−0.75 −0.50 −0.25 0.00 0.25 0.50 0.75 1.00
SHAP value
Against HF ⟵
⟶ In favor of HF
High
Feature value
Low

(d)

Table S2 - Patient characteristics for false positive cases and false negative cases

	**False positives** *n* = 19	**False negatives** *n* = 7
**CT findings**		
Ground-glass opacities, *n* (%)	8 (42.1)	4 (57.1)
Interlobular thickening, *n* (%)	2 (10.5)	4 (57.1)
Interlobar thickening, *n* (%)	5 (26.3)	1 (14.3)
Crazy paving, *n* (%)	0 (0)	0 (0)
Consolidations, *n* (%)	3 (15.8)	0 (0)
Pleural effusion, *n* (%)	13 (68.4)	3 (42.9)
Atelectasis, *n* (%)	9 (47.4)	1 (14.3)
Increased vascular diameter, *n* (%)	3 (15.8)	3 (42.9)
Peribronchial cuffing, *n* (%)	2 (10.5)	1 (14.3)
Enlarged heart, *n* (%)	5 (26.3)	4 (57.1)
Emphysema, *n* (%)	7 (36.8)	1 (14.3)
Pneumonic infiltrates, *n* (%)	9 (47.4)	3 (42.9)
**Diagnoses by expert panels**		
Acute pulmonary disease by pulmonologists, *n* (%)	14 (73.7)	1 (14.3)
Radiologic AHF according to research radiologist 1, *n* (%)	0 (0)	1 (14.3)
Radiologic AHF according to research radiologist 2, *n* (%)	0 (0)	2 (28.6)
Cardiologist-AHF, *n* (%)	1 (5.3)	7 (100)
Cardiologist-AHF with significant acute pulmonary disease, *n* (%)	2 (10.5)	0 (0)
Echo-BNP AHF, *n* (%)	8 (42.1)	7 (100)
**Signs and symptoms at admission**		
Systolic blood pressure (mmHg), mean (SD)	139 (26.5)	153 (23.9)
Orthopnea, *n* (%)	9 (47.4%)	4 (57.1%)
Bilateral pedal oedemas, *n* (%)	7 (36.8%)	1 (14.3%)
Bilateral rales, *n* (%)	7 (36.8%)	3 (42.9%)
**Laboratory data**		
LVEF, median [IQR]	58.0 [50.5;60.0]	40.0 [31.0;47.5]
NT-proBNP (pg/mL), median [IQR]	3336 (2570)	3113 (1465)
**Echocardiographic phenotype**		
Reduced LVEF≤40%, *n* (%)	2 (10.5)	3 (42.9)
Mildly reduced LVEF from 41-49%, *n* (%)	1 (5.26)	1 (14.3)
LVEF ≥ 50% with diastolic dysfunction, *n* (%)	9 (47.4)	2 (28.6)
Severe valve disease, *n* (%)	1 (5.26)	1 (14.3)
None of the above, *n* (%)	6 (31.6)	0 (0)
**History of heart failure**	7 (36.8)	3 (42.9)

CT findings were identified as the consensus by the two research radiologists. False positive was defined as AI predicted AHF using the optimal threshold maximizing the combination of sensitivity and specificity, but without radiological signs of AHF by the radiology report. False negative was defined as no AI predicted AHF, but AHF was identified in the radiology report. *AHF* Acute heart failure, *BNP* Brain natriuretic peptide, *Echo* Echocardiography, *LVEF* Left ventricular ejection fraction, *NT-proBNP* N-terminal pro-B-type natriuretic peptide.

Appendix 1 - Overview of the primary and secondary outcomes

Appendix content index

Section	Page No in appendix
**Primary outcome** – Radiology report AHF	32
**Secondary outcome** – Cardiology assessment of AHF<br>1) Cardiologist-AHF<br>2) Cardiologist-AHF with significant acute pulmonary disease<br>3) Echo-BNP AHF	33
**Secondary outcome** – Radiology Assessment of AHF<br>4) Research radiologist, Reader 1<br>5) Research radiologist, Reader 2<br>6) Research radiologists consensus AHF	34

PRIMARY OUTCOME – Radiology Report AHF by the on-call radiologists

Radiology report AHF was defined as:
The on-call radiologist evaluated images with access to all prior radiology and medical records. The final clinical radiology report used as primary outcome diagnosis was always confirmed by a second senior radiologist. Different on-call clinical radiologists (*n* = 20) were responsible for the reference diagnosis.

SECONDARY OUTCOMES – Three radiological and three cardiological AHF diagnoses

Cardiology diagnoses were defined as:

1) **Cardiologist-AHF**: Adjudicated by two cardiologists (and a third in case of disagreement) according to a modified version of the 2017 cardiovascular and stroke endpoint definitions for clinical trials consensus report[4, 7, 14, 24, 25] based on the following information:
    - Review of echocardiography images with evidence of abnormal structure, function, and LV filling pressures (grade II+III)
    - Review of medical record information including, history, blood samples, but without direct evaluation of radiology images
    - Excluding patients with a confirmed significant pulmonary disease as a possible cause of acute dyspnea.
2) **Cardiologist-AHF with significant acute pulmonary disease**: Similar criteria as for the principal AHF diagnosis, but also including patients with acute significant pulmonary disease as confirmed by pulmonologists. Any chronic or acute pulmonary disease was adjudicated by two specialists in pulmonary medicine, evaluating the medical record including blood samples, microbiological samples from sputum, medication, and radiology after patient discharge.

3) **Echo-BNP AHF:** This **operator-independent** diagnosis was designed to serve as a more objective comparator. This helped mitigate potential bias from medical record reviews that may have been influenced by radiology imaging. It was defined as **the presence of all four objective criteria**:

- echocardiographic abnormal structure or function; left ventricular ejection fraction (LVEF) ≤ 40%, LVEF 41-49%, LVEF >=50% with diastolic dysfunction or severe valve disease [reference #4];
- NT-proBNP >300 pg/ml [reference #4];
- signs of elevated left ventricle filling pressure on echocardiography (grade II+III) [reference #25];
- administration of loop diuretics orally or intravenously during admission or at discharge.

Radiology diagnoses were defined as:

4) **Research radiologist, Reader 1**: The patient exhibits radiological signs of AHF as assessed by thoracic expert radiologist (reader 1), including patients with radiological signs of AHF both with and without simultaneous acute pulmonary disease (Likert 4 or 5). The radiologist evaluated current radiology images, blinded to all clinical data, previous radiology images and echocardiography. The only clinical information provided was that the patient was part of the FACTUAL study and that the image therefore was obtained due to dyspnea.
5) **Research radiologist, Reader 2**: Assessment was conducted identically by Reader 2, following the same blinded evaluation protocol as Reader 1.
6) **Research radiologists consensus AHF**: AHF was defined as the agreement between Reader 1 and Reader 2, with both assigning a score of **4 or 5** on the 5-point Likert scale — indicating a diagnosis of "probably AHF," with or without concurrent acute pulmonary disease.

The 5-point Likert scale:

1 = Very unlikely

2 = Somewhat unlikely

3 = Neutral

4 = Somewhat probable (signs of radiological AHF with simultaneous acute pulmonary disease)

5 = Very probable (radiological AHF signs only)

AHF Acute heart failure.
